# The Effects of Environmental Adversities on Human Neocortical Neurogenesis Modeled in Brain Organoids

**DOI:** 10.3389/fmolb.2021.686410

**Published:** 2021-06-24

**Authors:** Kseniia Sarieva, Simone Mayer

**Affiliations:** ^1^ Hertie Institute for Clinical Brain Research, University of Tübingen, Tübingen, Germany; ^2^ International Max Planck Research School, Graduate Training Centre of Neuroscience, University of Tübingen, Tübingen, Germany

**Keywords:** brain organoid, environmental programming, neurogenesis, corticogenesis, neural stem/progenitor cells

## Abstract

Over the past decades, a growing body of evidence has demonstrated the impact of prenatal environmental adversity on the development of the human embryonic and fetal brain. Prenatal environmental adversity includes infectious agents, medication, and substances of use as well as inherently maternal factors, such as diabetes and stress. These adversities may cause long-lasting effects if occurring in sensitive time windows and, therefore, have high clinical relevance. However, our knowledge of their influence on specific cellular and molecular processes of *in utero* brain development remains scarce. This gap of knowledge can be partially explained by the restricted experimental access to the human embryonic and fetal brain and limited recapitulation of human-specific neurodevelopmental events in model organisms. In the past years, novel 3D human stem cell-based *in vitro* modeling systems, so-called brain organoids, have proven their applicability for modeling early events of human brain development in health and disease. Since their emergence, brain organoids have been successfully employed to study molecular mechanisms of Zika and Herpes simplex virus-associated microcephaly, as well as more subtle events happening upon maternal alcohol and nicotine consumption. These studies converge on pathological mechanisms targeting neural stem cells. In this review, we discuss how brain organoids have recently revealed commonalities and differences in the effects of environmental adversities on human neurogenesis. We highlight both the breakthroughs in understanding the molecular consequences of environmental exposures achieved using organoids as well as the on-going challenges in the field related to variability in protocols and a lack of benchmarking, which make cross-study comparisons difficult.

## Introduction

Environmental adversities acting on the embryo or fetus may shape brain development and predispose it to neurological and psychiatric diseases later in life. Such developmental alterations upon environmental exposures are called environmental programming. The environmental exposures include maternal factors as well as external ones, such as viral infections (Faa et al., 2016; Meyer, 2019). An example of how maternal factors may affect fetal neurodevelopment is maternal malnutrition, which correlates with multiple neurodevelopmental deficits in the offspring, including changes in memory formation and motor function (Faa et al., 2016). Neurodevelopmental deficits often involve higher cognitive functions, suggesting alterations in neocortical development. The mechanisms underlying environmental programming have been difficult to investigate for several reasons. On the one hand, the correlations were found in epidemiological studies and require further perturbation experiments for causal inference. On the other hand, human intrauterine development is mostly inaccessible for perturbation studies or research into the cellular and molecular effects of environmental adversities.

The *in utero* development of the human nervous system includes several phases. It starts with neural induction and the formation of the neural tube, followed by the expansion of neural stem cell (NSC) pools, the subsequent generation of neurons (neurogenesis) and glial cells (gliogenesis), and, finally, the establishment and maturation of neuronal circuits. In this review, we focus on two phases: NSCs proliferation and neurogenesis in the embryonic and fetal neocortex (roughly, postconceptional weeks (PCW) 6–27) (Silbereis et al., 2016).

Mammalian model organisms have proven highly valuable to reveal mechanisms of NSCs proliferation and neurogenesis as both the neocortex itself and the intrauterine development are characteristics of mammals (Cárdenas and Borrell, 2020). Especially rodent models have catalyzed our understanding of the spatial and temporal dynamics of neocortical development. Additionally, rodents have been most extensively used to study various aspects of the environmental effects on neurogenesis. For instance, murine studies have allowed researchers to characterize the effects of maternal nicotine exposure on fetal neurogenesis (Aoyama et al., 2016). However, in contrast to the human brain, the murine brain is lissencephalic and has a restricted repertoire of NSCs (Uzquiano et al., 2018). The number of divisions that murine NSCs undergo before differentiation is also lower than in human NSCs (Lui et al., 2011). Additionally, mice as a species have different cellular dynamics, partially due to the relative instability of murine proteins compared to human ones (Rayon et al., 2020). This translates into faster cellular differentiation (Rayon et al., 2020): neurogenesis in mice is completed in a relatively short time frame (embryonic day (E) 9–14) (La Manno et al., 2020) whereas in humans it takes almost 4.5 months of pregnancy (Silbereis et al., 2016). Altogether, the differences between mouse and human brain development call for alternative model systems that recapitulate human-specific traits of neocortical development more closely. Using gyrencephalic animals, including ferret and non-human primates, is a solution to the differences between human and rodent brain development. These species have a larger variety of NSCs types than the mouse, specifically, outer radial glia (see detailed description below) (Lui et al., 2011). Additionally, primates partially recapitulate the long neurogenic period characteristic for humans (Kornack and Rakic, 1998). However, limitations for the use of these animals exist from both ethical and experimental perspectives. For instance, the experiments are difficult to scale, thus limiting experimental designs to those showing large effect size.

In some rare cases, human fetal tissue is accessible for experimentation. It provides valuable information on some species-specific mechanisms driving neurogenesis as well as particular architectural, cellular, and molecular aspects of human neurodevelopment (Pollen et al., 2015; Fiddes et al., 2018; Mayer et al., 2019; Xing et al., 2020). Specifically, human neocortical organotypic slice cultures were used to study mechanisms of Zika virus-induced microcephaly (Onorati et al., 2016). Studies with human fetal tissues pose similar ethical and experimental difficulties as studies on non-human primates. One of the main experimental challenges is that fetal neocortical organotypic slice cultures can only be maintained for several days or up to three weeks using an automated platform precluding long-term interventions (Linsley et al., 2019).

Various *ex vivo* systems helped to partially overcome existing limitations. For instance, primary human NSCs were used to characterize mitotic defects upon Zika virus infection (Onorati et al., 2016). However, larger experimental flexibility was achieved with the development of protocols for neural differentiation from pluripotent stem cells (PSCs), both induced PSCs and embryonic stem cells (Shi et al., 2012). Over the past years, the growing efficiency of the existing differentiation protocols allowed for large-scale screens for various neurotoxins (Pei et al., 2016; Schmidt et al., 2017). However, two-dimensional (2D) cultures lack cytoarchitectural properties of the developing brain. This limitation has recently been overcome with the development of the PSCs-derived 3D systems, so-called brain organoids (Kadoshima et al., 2013; Lancaster et al., 2013). Brain organoids combine the strengths of both *in vivo* and *in vitro* approaches: they recapitulate the cellular composition and the cytoarchitecture of the human neocortex while preserving the scalability and experimental tractability of *in vitro* approaches. These features have made brain organoids an attractive model system for studying the effects of environmental adversities on human neurodevelopment.

**FIGURE 1:**
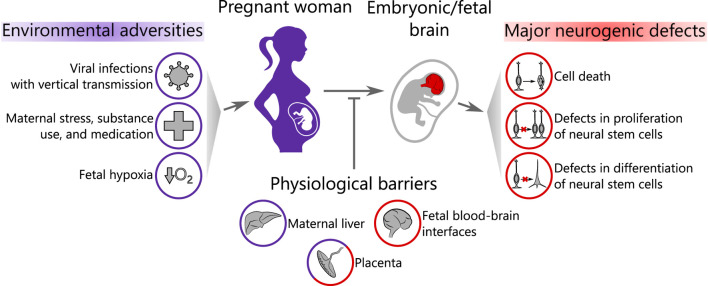
Scope of the review. Environmental adversities affect the pregnant woman and may pass through a series of biological barriers to the embryonic/fetal brain causing neurogenic defects. Purple color represents the maternal compartment and red color corresponds to the embryo/fetus.

In this review, we provide an overview of the recent advances in using brain organoids to study environmental programming on human neocortical neurogenesis. We first describe how brain organoids mimic features of human neocortical development during stages of neurogenesis. Next, we will introduce the biological barriers that various compounds and infectious agents have to cross before entering the fetal brain *in vivo*. We will then focus on the effects of several major groups of environmental adversities on neurogenesis, which have recently been investigated using brain organoids as a model system: viral infections with vertical transmission from mother to fetus, maternal stress, substance use and medication, and, finally, fetal hypoxia (Figure 1). We conclude by discussing experimental limitations in this relatively young field and potential routes for its further development.

## Major Populations of Cortical Neural Stem Cells Can be Found in Brain Organoids

**FIGURE 2:**
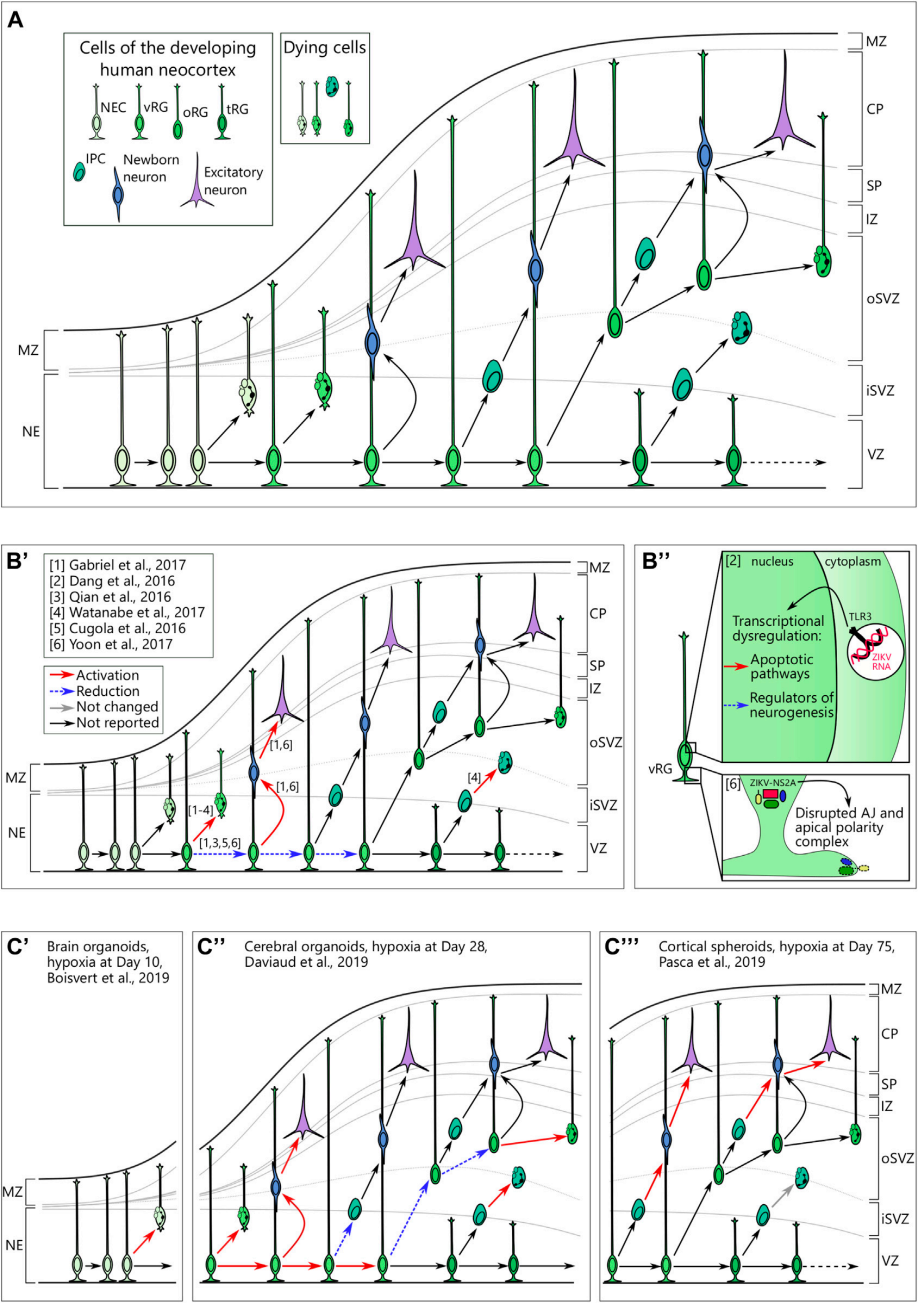
**(A)** Cellular view of the normal human neocortical development. Human neocortical development starts from neuroepithelial cells (NEC), which further transform to ventricular radial glia (vRG). Both NEC and vRG have nuclei next to the ventricle (neuroeplithelium (NE) and ventricular zone (VZ), respectively) and a basal process spanning through the cortical wall and marginal zone (MZ) to reach the pial surface. vRG can either divide symmetrically to generate two vRGs or differentiate to produce excitatory neurons or basal neural progenitor cells. Basal progenitor cells reside in the inner (iSVZ) and outer (oSVZ) subventricular zones and include intermediate progenitor cells (IPC) and outer radial glia (oRG). Both types of basal neural progenitor cells can self-amplify or differentiate into excitatory neurons. Later in development, vRG lose connection to the pial surface and transform into truncated radial glia (tRG) while still generating basal progenitors and excitatory neurons. Newborn neurons of different origins migrate along the radial fibers through the intermediate zone (IZ) and subplate (SP) and take their place in the cortical plate (CP). A low level of cell death in neural cells is normal over neurogenesis. **(B)** Brain organoids infected with Zika virus reveal cellular mechanisms of microcephaly. B’. Multiple studies suggest preferential infection of neural progenitor cells over neurons. Infection in vRG leads to the apoptotic cell death, attenuation of proliferation as well as shift to direct neurogenesis. **(B’’)** upper panel. Zika virus (ZIKV) activates Toll-like receptor 3 in vRGs that leads to transcriptional deregulation with activation of apoptotic pathways and inhibition of regulators of neurogenesis. Adapted from Dang et al. (2016). **(B’’)** lower panel. NS2A, a protein of ZIKV envelop, binds the components of adherens junctions (AJ) in the cytoplasm and prevents them from forming functional AJ and apical polarity complex. Reprinted from Yoon et al. 2017 with permission from Elsevier. Copyright (2017). **(C)** Hypoxic exposure in brain organoids at different time points results in distinct defects on the cellular level. **(C’)** Hypoxic exposure (1% O_2_, 72 h) at day 10 of organoid differentiation results in the increased cell death, presumably, of NECs. **(C’’)** When hypoxia (3% O_2_, 24 h) is applied to brain organoids at day 28, it results in the immediate cell death across the cortical wall followed by proliferation in vRG. Subsequently, vRG tend to differentiate into neurons at the expense of generation of both IPCs and oRG, which results in the decreased number of these cells 14 days after hypoxic exposure. **(C’’’)** Cortical spheroids that are exposed to hypoxia (1% O_2_, 48 h) at day 75 of differentiation show decreased numbers of IPCs resulting from premature differentiation but not from cell death in these cells.

Throughout mammalian brain development, specific populations of stem cells sequentially produce a vast diversity of neurons in a predefined order (Telley et al., 2019; Sagner et al., 2020). Neocortical development in humans largely follows the same logic (Figure 2A). The first population of NSCs in the telencephalon emerges at neurulation (around PCW4) and is named neuroepithelial cells (NEC). These cells divide predominantly in a symmetrical manner to expand the pool of proliferating cells (Subramanian et al., 2017). Upon replacement of tight junctions with adherens junctions at the apical polarity complex, NECs transform to neural precursor/progenitor cells (NPC) of the ventricular zone, the so-called ventricular or apical radial glia (vRG) cells (Uzquiano et al., 2018). The cells with vRG properties emerge around PCW6-8 (Subramanian et al., 2017). They are capable of both symmetric and asymmetric divisions that either promote self-renewal of this population or generate transit-amplifying/intermediate progenitor cells (IPC) and newborn neurons (Noctor et al., 2004), respectively. vRG processes span from the ventricular to the pial surface and provide a scaffold for migrating neurons (Noctor et al., 2001). Starting from PCW10, vRGs give rise to a unique population of NPCs that is almost exclusively present in the neocortex of gyrencephalic mammals, the so-called outer or basal radial glia (oRG) (Smart et al., 2002; Fietz et al., 2010; Hansen et al., 2010; Reillo et al., 2011; Wang et al., 2011). oRGs are localized in the inner and outer subventricular zone (iSVZ, oSVZ) and predominantly divide asymmetrically to generate daughter oRG and an IPC or a neuron (Hansen et al., 2010; Betizeau et al., 2013). Later, around PCW15, vRG cells lose their connection to the pial surface and transform into truncated radial glial (tRG) cells, while still giving rise to excitatory neurons (Nowakowski et al., 2016b).

The first neurons of the developing neocortex, the so-called Cajal-Retzius cells, originate from the cortical hem as well as from other sources at the early stages of neurogenesis (Meyer, 2010). This heterogeneous neuronal population resides in the marginal zone (MZ) and controls migration and laminar positioning of the subsequent excitatory neurons through secretion of the extracellular matrix glycoprotein Reelin (Meyer, 2010). The excitatory neurons of layers II-VI are generated from RG cells as well as from IPCs in an inside-out manner in which deeper layers are generated before upper layers. Thus, layer VI neurons are being born and migrate to their final location first followed by layer V neurons and, finally, by layer II-IV neurons. A more detailed review on *in utero* human cortical neurogenesis can be found elsewhere (Lui et al., 2011; Taverna et al., 2014; Silbereis et al., 2016; Uzquiano et al., 2018).

Human PSC-derived brain organoids recapitulate both temporal and spatial components of the neocortical development. Cerebral organoids at days 10–15 after seeding show tight junctions at the apical side of the SOX2-positive neural rosettes reminiscent of NECs in the developing human brain (Benito-Kwiecinski et al., 2021). At week 5 of differentiation, cortical organoids possess mitotic NECs that are later replaced by vRGs (Subramanian et al., 2017). Both vRGs and oRGs and IPCs can be readily identified in the majority of cerebral and neocortical organoids protocols (Quadrato et al., 2017; Kanton et al., 2019; Pollen et al., 2019; Velasco et al., 2019). Additionally, NSCs in brain organoids mimic the cell cycle characteristics of primate NSCs rather than murine NSCs (Kornack and Rakic, 1998; Mora-Bermúdez et al., 2016). Furthermore, the differentiation trajectory from stem cell-like toward neuronal identity correlates between many organoid protocols and human fetal neocortex (Camp et al., 2015; Pollen et al., 2019; Tanaka et al., 2020) as well as temporal dynamics of this differentiation (Tanaka et al., 2020). This results in the generation of both lower (V-VI) and upper (II-IV) layer excitatory neurons in a sequential manner. The separation of the markers of these neuronal layers reflects maturation of the organoids and it has become partially feasible with the improvement of organoid protocols (Qian et al., 2020).

In this review, we summarize studies employing different types of brain organoids as a model system. The so-called “cerebral organoids” are generated according to the protocol by Lancaster et al. (2013) in its original or a modified version. At the first stages of this protocol, PSCs are seeded at densities that allow the formation of embryoid bodies, which are further subjected to neural induction at day 6 after seeding. From day 11 in culture, a change in media composition ensures neuronal differentiation and maturation (Lancaster et al., 2013). As a result, cerebral organoids develop a large variety of regional identities of the brain, including neocortical, choroid plexus and hindbrain (Lancaster et al., 2013; Lancaster and Knoblich, 2014). This protocol is therefore referred to as “unguided” (Qian et al., 2019). In order to specifically induce the formation of a particular brain region, organoids may be subjected to patterning factors, the so-called morphogens. Such organoid differentiation protocols are referred to as “guided” protocols (Qian et al., 2019). Morphogens used to guide dorsal forebrain identity include TGF-β, Wnt and BMP pathway inhibitors (Kadoshima et al., 2013; Paşca et al., 2015; Velasco et al., 2019) while BMP and Wnt pathway activators are required for induction of choroid plexus identity (Pellegrini et al., 2020b). A more detailed review of the different organoid differentiation protocols can be found elsewhere (Qian et al., 2019; Khakipoor et al., 2020).

## Maternal and Fetal Barriers Protect Fetal Brain From the Effects of Environmental Adversities

Although human brain organoids closely recapitulate human neocortical development, their use for modeling the effects of environmental adversities on fetal neurodevelopment requires validation. Specifically, the developing fetal brain is protected from the environment by multiple barriers that are absent in an *in vitro* setting. These barriers include maternal protective mechanisms, such as inactivation of xenobiotics in the liver, as well as placental barrier and fetal blood-brain interfaces (Dorman et al., 2001; Ek et al., 2012; Tetro et al., 2018).

The placenta plays a dual role in fetal neurodevelopment. On the one hand, it transports maternal hormones, nutrients and oxygen to the fetus (Tetro et al., 2018). On the other hand, it protects the fetus from xenobiotics and other potentially harmful compounds derived from maternal blood through the selective influx of various compounds from maternal to fetal circulation, enzymatic inactivation of xenobiotics, and their efflux from fetal to maternal blood (Tetro et al., 2018). Trophoblast and capillary endothelial cells take over the main barrier function (Tetro et al., 2018).

Fetal blood-brain interfaces include the blood-brain barrier (BBB), blood-cerebrospinal fluid (CSF) and meningeal barrier as well as a fetus-specific brain-CSF barrier (Ek et al., 2012; Strazielle and Ghersi-Egea, 2013; Goasdoué et al., 2017). The primary barrier function of the fetal BBB is performed by the endothelial cells and is established upon vascularization of the neocortex (Ek et al., 2012). Endothelial cells express tight junction proteins starting from PCW8 (Møllgård and Saunders, 1975) and protect the fetal brain from protein entry starting from PCW10 (Virgintino et al., 2000). It is not clear when fetal endothelial cells gain similar transport properties as the adult ones (Ek et al., 2012). Fully functional endothelial cells are permissive to gases, lipophilic molecules, and a subset of small molecules (<400 Da) (Ross et al., 2020). Additionally, they are capable of carrier-mediated, receptor-mediated, and active transport (Ross et al., 2020).

The fetal blood-CSF barrier is formed by epithelial choroid plexus (ChP) cells. ChP cells are bound together with tight junctions located apically as early as PCW8 (Møllgård and Saunders, 1986). Interestingly, the blood-CSF barrier both in fetal development and adulthood is permissive to plasma protein entry to the CSF (Strazielle and Ghersi-Egea, 2013). The barrier and transport functions of the ChP complement its synthetic and secretory function. Embryonic CSF produced by ChP cells can maintain proliferative divisions in NPCs in the *ex vivo* setting through a combination of instructive cues, including insulin-like growth factor 2 (IGF2) in rats (Lehtinen et al., 2011). Interestingly, both barrier function of the ChP and its secretome can be recapitulated *in vitro* with ChP organoids (Pellegrini et al., 2020b).

Like ChP and endothelial cells, cells of the arachnoid that form the meningeal barrier also possess tight junctions (Ek et al., 2012). Additionally, the fetal brain contains a specific barrier that is not found in adults, the so-called brain-CSF barrier (Møllgård et al., 1987; Whish et al., 2015). It is formed by the “strap” junctions between the vRGs before their differentiation to ependymal cells (Møllgård et al., 1987; Whish et al., 2015). This brain-CSF barrier contains components of adherens and tight junctions and does not allow molecules as small as 268 Da to enter brain parenchyma at E17 in mice (Whish et al., 2015). Together, these studies suggest that the fetal brain is protected from entry of large and/or water-soluble molecules unless specific transport mechanisms exist.

Despite advances in understanding barrier properties at the tissue level, less is known about molecular transport and xenobiotic efflux machinery in the developing brain. However, recent studies indicate that the major efflux pump of the BBB, P-glycoprotein (P-gp), is expressed as early as PCW8-10 in fetal capillaries and increases with gestational age and postnatally (Schumacher and Mollgård, 1997; Daood et al., 2008; Lam et al., 2015; De Trizio et al., 2020). P-gp expression in the fetal BBB is altered by glucocorticoids (Petropoulos et al., 2010) and selective serotonin reuptake inhibitors (Bhuiyan et al., 2012). Thus, environmental adversities may not only affect the developing brain by reaching it but also by changing the barrier functions that protect it.

## Viral Infections With Vertical Transmission From Mother to Fetus

There is a limited number of infectious diseases that can be transmitted from mother to embryo or fetus through either transplacental or intrauterine routes including the well-known ToRCH pathogens (toxoplasmosis, rubella, cytomegalovirus, herpes simplex virus, and others). It has been difficult to study the effects of these pathogens on neurodevelopment in animal models due to the high species specificity of the infectious agents. The applicability of brain organoids to studying these diseases was first shown during the Zika virus outbreak that led to microcephaly cases predominantly in Brazil starting in 2015. Since then, brain organoids have been used to model various viral infections and even to test and develop therapeutic strategies (Watanabe et al., 2017; Xu et al., 2019).

### Zika Virus Causes Massive Cellular Death in Neural Precursor/Progenitor Cells as Well as Structural Disorganization of Proliferative Zones

The recent breakout of Zika virus showed multiple abnormalities in the developing fetuses of infected mothers. Indeed, the first-trimester human placenta is not only permissive to the viral infection but also supports its replication (Pereira, 2018; Pique-Regi et al., 2020). Among other defects, Zika virus induces severe microcephaly in fetuses through preferential infection of NPCs as seen in primary tissue (Onorati et al., 2016; Retallack et al., 2016). The potential cause of the microcephalic phenotype was replicated in induced PSC-derived neurospheres where Zika virus showed a productive infection of NPCs and caused severe size defects (Cugola et al., 2016). Following this initial finding, brain organoids infected by Zika virus showed impaired growth in multiple studies (Dang et al., 2016; Garcez et al., 2016; Watanabe et al., 2017). The restricted growth was accompanied by increased cell death, and disrupted proliferative zones and cortical layering (Qian et al., 2016; Wells et al., 2016; Watanabe et al., 2017) (Figure 2B’). Although microcephaly can be readily detected in a murine model of Zika virus infection (Cugola et al., 2016), brain organoids proved to be a versatile experimental platform. Particularly, several independent studies have shown tropism of the Zika virus for oRGs (Qian et al., 2016; Watanabe et al., 2017). Moreover, using brain organoids, several studies revealed the molecular mechanisms of Zika virus toxicity in NPCs. Infected NPCs showed a perturbed centrosomal structure, which was accompanied by abnormal division planes and premature differentiation (Gabriel et al., 2017). Another study indicates the specific activation of the immune receptor Toll-like receptor 3 (TLR3) upon Zika virus infection as the upstream mechanism of premature differentiation (Dang et al., 2016) (Figure 2B’’). Activation of the anti-viral immune response in NPCs was corroborated in another study (Watanabe et al., 2017) suggesting the robustness of the experimental results between the different studies. Moreover, Watanabe and colleagues tested potential treatment strategies (Watanabe et al., 2017). One antibiotic, duramycin, targeted the viral lipid membrane to prevent its interaction with the suggested cell entry receptors for Zika virus AXL, TYRO3, MER, and TIM1 and partially rescued the effect of Zika infection on organoids (Watanabe et al., 2017). However, another study showed that knockout of AXL alone, the presumptive principal cell entry receptor for Zika virus (Nowakowski et al., 2016a), did not attenuate pathological phenotype in cerebral organoids upon Zika virus infection (Wells et al., 2016).

Among other potential mechanisms that were validated both in murine and brain organoid models, there was disruption of adherens junctions in the vRGs leading to premature differentiation and, potentially, to aberrant newborn neuron migration along radial fibers (Yoon et al., 2017) (Figure 2B’’’). Brain organoid studies also hinted at the mechanism of infection, showing tropism of viral particles toward aquaporin-1 (AQP1)-positive cells which may have represented ChP cells (Watanabe et al., 2017). A recent meta-study on modeling Zika virus infection in brain organoids has shown a high correlation between the results from different research groups and employing different organoid differentiation protocols (Sutarjono, 2019). The consensus is that Zika virus reduces proliferation in NPCs, induces selective cell death in NPCs, and, finally, decreases the size of the organoid (Sutarjono, 2019). Later, 3D image analysis of the morphology of the organoids infected by Zika revealed a decreased number of VZ-like areas as well as the number of SOX2-positive RGs and TBR1-positive layer VI neurons (Albanese et al., 2020). However, the same analysis found a decrease in size of ventricle-like cavities, which is inconsistent with clinical observations of ventriculomegaly characteristic to Zika virus-induced microcephaly (De Fatima Vasco Aragao et al., 2016; Albanese et al., 2020). Ventriculomegaly in humans is associated with the massive loss of neural tissue (De Fatima Vasco Aragao et al., 2016), which is recapitulated in brain organoids. Altogether, these discrepancies highlight the necessity to critically assess of features of human brain development that can be modeled with brain organoids.

In summary, brain organoids have helped to reveal the particular vulnerability of the NPCs to the Zika virus infection, the cell tropism and molecular alterations that may explain microcephaly in patients. Finally, during the emergence of Zika virus-related studies, it was shown that brain organoids can be used as a platform for drug screening (Watanabe et al., 2017; Xu et al., 2019).

### Cytomegaloviral Infection of Neural Precursor/Progenitor Cells Leads to Severe Microcephaly in the Developing Fetus

Cytomegalovirus (CMV) is a threat to the developing human embryo or fetus upon transmission from the mother. Primary CMV infection during pregnancy results in the 40% chance of vertical transmission (Enders et al., 2011). A recent study has shown expression of CMV cell entry receptors in placental cells (Pique-Regi et al., 2020). The syncytiotrophoblast cells, which form the fetal part of placental barrier, are permissive to viral infection and replication in the first and second trimester (Fisher et al., 2000). This may lead to viral infection of multiple cell types within the embryo, including NSCs (Baggiani et al., 2020). Infection of murine embryos with murine CMV showed that ventricular and subventricular zones are the most affected (Tsutsui et al., 2005). *In vitro*, CMV infection of primary human NSCs impaired their proliferation with expression of viral genes persisting in glial but not neuronal differentiation (Cheeran et al., 2005). Another study indicates impaired proliferation and neuronal differentiation as well as increased cell death in primary human NPCs upon CMV infection (Odeberg et al., 2006). Recent experiments employing human brain organoids showed impaired organoid growth after CMV infection mimicking clinical observations of microcephaly (Sun et al., 2020). Sun and colleagues further confirmed previous suggestions on the CMV cell entry route through PDGFRa and EGFR (Sun et al., 2020). Additionally, the authors found a partial colocalization of TBR2-positive IPCs with the CMV protein IE1 (immediate-early 1) (Sun et al., 2020). Furthermore, recently Brown and coworkers showed massive changes in the morphology of the organoids upon CMV infection, including the presence of regions of necrosis, cysts as well as an overall decrease in cellularity (Brown et al., 2019). Additionally, the authors noticed the disorganization of both Nestin and Tuj1-positive areas suggesting that the primary effect on NPCs also leads to disrupted neurogenesis (Brown et al., 2019). Employing 3D engineered neural tissue, various transcriptional programs were found to be differentially expressed as infection progressed (Cosset et al., 2015). Three days after infection, genes related to lipid metabolism were upregulated, followed by an upregulation of developmental genes and genes involved in inflammatory response at day 5 and 7 after infection, respectively. The authors found hardly any PAX6-positive cells to be infected (Cosset et al., 2015). In their hands, the infected population consisted of the cells adjacent to the neural rosettes and positive for doublecortin (Cosset et al., 2015), a marker of newborn neurons (Ayanlaja et al., 2017). Taken together, a plethora of studies in animal models, primary human NSCs and organoids converge on CMV primarily infecting NSCs with subsequent deficits in neuronal and glial differentiation thus mimicking clinical observations.

### *In Utero* Herpes Simplex Virus Infection Leads to Massive Neurodevelopmental Abnormalities

The herpes simplex virus (HSV) family consists of two members, HSV-1 and HSV-2. According to the WHO data for 2016, HSV-1 is carried by ∼64% of the world population under 50 years old whereas the prevalence of the HSV-2 infection is ∼13% among people between 15 and 49 years old (James et al., 2020). The majority of cases of HSV vertical transmission from mother to child happens intrapartum leading to so-called neonatal herpes, which can cause severe complications and fatality of ∼60% if left untreated (Looker et al., 2017). Intrauterine infection is rare and controversies remain about the route of transmission from mother to fetus (Avgil and Ornoy, 2006; Avila et al., 2020). Intrauterine infection causes severe multiorgan abnormalities that are often lethal (Avgil and Ornoy, 2006). Abnormalities may include skin scarring, microcephaly, hydranencephaly, and ophthalmic manifestations (Hutto et al., 1987). In adults, neurons of both the central and peripheral nervous system can be infected by the HSV family with possible viral quiescence and reactivation later in life (Berger and Houff, 2008). The mechanisms of latency and reactivation remain poorly understood due to the human-specific nature of the infection leading to controversial results coming from animal experiments (D’Aiuto et al., 2019). Recently, an organoid differentiation strategy that is distinct from the original PSC-derived organoid protocols was employed to study the mechanisms of HSV-1 infection, quiescence, and reactivation in neuronal cells (D’Aiuto et al., 2019). Specifically, induced PSCs were differentiated into NPCs in adherent culture followed by the differentiation of the NPCs in a 3D environment (D’Aiuto et al., 2019). While modeling quiescent infection in a 3D environment is important for better understanding of the mechanisms of the host-virus interaction, the results obtained for the lytic infection in brain organoids may also have implications from the neurodevelopmental perspective. Specifically, the authors showed the degeneration of neurites as well as cell-cell fusion followed by generation of neuronal syncytia (D’Aiuto et al., 2019). In concordance with this finding, syncytia formed by various neural cells can also be observed in the murine organotypic slice cultures upon HSV-1 infection (Ecob-Johnston and Whetsell, 1979). It has been suggested that HSV use cell-cell fusion to avoid the “cell-free” spread of viral particles and thus evade interaction with host immune system (Carmichael et al., 2018). It remains to be investigated whether the intrauterine HSV infection-associated neural damage can be attributed to cell-cell fusion. Another study employed cerebral organoids at day 15 of differentiation to study the effects of HSV-1 infection (Qiao et al., 2020). The authors found a relative decrease of Nestin and SOX2 expression, which was accompanied by a decrease of the expression of Tuj1 and in the thickness of the cortical plate-like structure (Qiao et al., 2020). However, the authors did not report colocalization of the cell type markers with viral proteins, therefore no cell type-specific assessment of the phenotype induced by HSV-1 exposure can be performed (Qiao et al., 2020). The early time point in organoid differentiation suggests that HSV-1 may have affected NSCs. This hypothesis has been corroborated by a recent preprint, which finds that HSV-1 infection resulted in the highest viral load in NPCs in the cerebral organoids enriched for neocortical cellular identities at day 60 of differentiation (Lancaster et al., 2017; Rybak-Wolf et al., 2021). Cell type-specific differential gene expression analysis showed upregulation of the cellular stress- and infectious disease-associated genes in both RGs and IPCs 3 days after infection (Rybak-Wolf et al., 2021). Additionally, IPCs showed a downregulation of the pathways associated with cell division (Rybak-Wolf et al., 2021). Apart from cell type-specific changes following HSV-1 infection, the authors demonstrated a global increase in poly(A) tail lengths and preferential choice of the distal 3’ adenylation site of mRNA molecules (Rybak-Wolf et al., 2021). Both measurements may be interpreted as restructuring of the translational machinery to favor viral replication but not host translation and mRNA turnover (Rybak-Wolf et al., 2021). Altogether, the results of these studies suggest that *in utero* HSV-1 infection may affect both NSCs and neurons and explain some of the clinical manifestations, such as microcephaly.

### SARS-CoV-2 Appears to Cross Placental Barrier and Infect Particular Types of Neural Cells During Fetal Development

Clinical observations from adult patients infected with the novel SARS-CoV-2 suggest that the disease may have neurological manifestations (Khan and Gomes, 2020). Although the mechanisms of neural damage in adults are of great clinical importance, the potential damage to the fetal brain during *in utero* development upon maternal infection should also be investigated. Indeed, several reports suggest transplacental transmission of the novel coronavirus from mother to fetus (Algarroba et al., 2020; Ashary et al., 2020; Hosier et al., 2020; Patanè et al., 2020; Vivanti et al., 2020) while others provide evidence against this possibility (Pique-Regi et al., 2020). The prevailing opinion, however, is that the two major SARS-CoV-2 cell entry mediators (angiotensin-converting enzyme 2, ACE2, and the S protein priming protease TMPRSS2) are expressed in the syncytiotrophoblasts cells and the extravillous trophoblasts during the first and the second trimester of pregnancy, respectively, (Ashary et al., 2020) and support virus entry into these cells (Hosier et al., 2020; Patanè et al., 2020). Moreover, bulk expression of both ACE2 and TMPRSS2 decreases in the human placenta as pregnancy progresses, showing a particular vulnerability of the fetus during the first half of gestation (Bloise et al., 2020). After crossing the placental barrier, SARS-CoV-2 can be found in the umbilical cord at midgestation (Hosier et al., 2020) and potentially reach the fetal brain through infection of the ChP as shown in ChP organoids (Jacob et al., 2020; Pellegrini et al., 2020a). In addition to the potential entry of viral particles into the brain, infection of ChP *per se* results in its severe malfunctioning where synthetic, secretory, barrier and transport functions are affected (Jacob et al., 2020). If ChP is permissive to SARS-CoV-2 brain entry, the question is which cell types within the brain may be affected by the virus. Several brain organoid studies show infection of NSCs (Mesci et al., 2020; Song et al., 2020; Zhang et al., 2020), neurons (Jacob et al., 2020; Mesci et al., 2020; Ramani et al., 2020; Song et al., 2020; Zhang et al., 2020; Andrews et al., 2021), and even astrocytes (Jacob et al., 2020; Mesci et al., 2020; Andrews et al., 2021) by the novel coronavirus. Considering neurogenesis, the next focus will be on the potential infection of NSCs and newborn neurons. Reports suggest tropism of SARS-CoV-2 to NPCs with viral proteins colocalizing with SOX2-positive neural rosettes (Song et al., 2020) and NPC marker Nestin (Mesci et al., 2020; Zhang et al., 2020). However, other reports do not support this observation (Andrews et al., 2021; Ramani et al., 2020). This discrepancy cannot be readily attributed to the protocol for organoid differentiation, their age, incubation time with the virus and the viral dose (Table 1) highlighting the need for further corroboration by independent studies with larger numbers of replications (see also *Brain Organoid Models Have Started to Reveal Cellular and Molecular Mechanisms of Environmental Insults* and *Technological Advances Drive Progress in Brain Organoid Research and Increase Interpretability Across Studies*). Interestingly, some studies report apoptotic cell death not only in the infected cells but also in the non-infected neighboring cells within brain organoids (Mesci et al., 2020; Ramani et al., 2020; Song et al., 2020). The proportion of apoptotic cells correlates with the proportion of SARS-CoV-2 infected cells suggesting non-cell autonomous toxicity of the virus, which may be attributed to the hypermetabolic state of infected cells and their ability to create a local hypoxic environment (Song et al., 2020). Additionally, the authors report shifts in cell type composition upon coronavirus infection with neuronal cells over- and transient progenitor cell underrepresented suggesting premature differentiation in the infected organoids (Song et al., 2020). Collectively, these studies indicate that SARS-CoV-2 infection may be potentially hazardous for the developing brain at peak neurogenesis due to permissiveness of the placental barrier and infection of ChP followed by viral entry into neural cells. The observed cell death and metabolic alterations upon SARS-CoV-2 exposure in brain organoids provide the potential for long-lasting neurodevelopmental defects in children after maternal infection. Additionally, systemic effects should also be investigated. Specifically, pregnant women are considered a risk group for developing severe symptoms upon SARS-CoV-2 infection (Allotey et al., 2020). Activation of maternal immune response has, in turn, been repeatedly correlated with an increased risk for neurodevelopmental disorders in the offspring (Meyer, 2019; Allswede et al., 2020).

**TABLE 1:** Comparison between different studies focusing on the cell type tropism of SARS-CoV-2.

Publication	Organoid protocol and age upon infection	Viral strain, time of exposure and MOI	Infection				Viral production			
			NSCs	Neurons	Astrocytes	Choroid plexus	NSCs	Neurons	Astrocytes	Choroid plexus
Jacob et al. (2020)	Cortical, age not reported	SARS-CoV-2 USA-WA1/2020, 8 h, MOI 0.1–0.05	N/A	+	+	+	−	−	−	+
Zhang et al. (2020)	Cerebral, Day 35	N/A, 72 h, N/A	+	+	N/A	N/A	+	N/A	N/A	N/A
Ramani et al. (2020)	Cerebral, Day 15 or 60	SARS-CoV-2 NRW-42, 2 days, MOI 1.8 × 10^−4^ for day 15, 8.8 × 10^05^ for day 60	−	+	N/A	N/A	−	−	N/A	N/A
Mesci et al. (2020)	Cortical, Day 52	SARS-CoV-2 isolated from a patient in Washington State, 1 week, MOI 2.5	+	+	+	N/A	N/A	N/A	N/A	N/A
Song et al. (2020)	Cerebral, Week 9	SARS-CoV-2 isolate USA-WA1/2020, 24 or 96 h, MOI 1	+	+	N/A	N/A	+	+	N/A	N/A
Andrews et al. (2021)	Cortical, Week 5, 10, 16 or 22	SARS-CoV-2 isolate USA-WA1/2020, 2 h, MOI 0.5	+	+	+	N/A	−	N/A	+	N/A

+, stands for positive; −, for negative; N/A, not investigated; MOI, multiplicity of infection.

## Maternal Stress, Medication, and Substance Use

### Glucocorticoids Disrupt Neural Precursor/Progenitor Cells Differentiation

Maternal stress has been repeatedly correlated with diverse neurodevelopmental abnormalities in the fetus with penetration into adolescence and adulthood (Krontira et al., 2020). However, human studies are mostly observational. This study design makes causal inference complicated due to the variety of confounding factors and the inability to analyze objective measurements of maternal stress (Dipietro, 2012). In this review, we will focus on the known molecular players that define maternal stress, the glucocorticoid (GC) hormones, and their effects on cortical neurogenesis.

GCs, being lipid-soluble molecules, readily cross the placental barrier and blood-brain interfaces (Benediktsson et al., 1997; Krontira et al., 2020). Although 50–90% of the main human GC hormone cortisol is inactivated in the human placenta by 11β-hydroxysteroid dehydrogenase type 2 (11βHSD2) (Mulder et al., 2002), chronic stress or inflammation, as well as maternal alcohol consumption, reduce placental activity of this enzyme (Howerton and Bale, 2012). It is, therefore, likely that maternal cortisol reaches the embryonic and fetal brain. In addition to natural glucocorticoids, their synthetic analogs (e.g. dexamethasone, betamethasone) are prescribed to pregnant women, for example to correct the risk (or the diagnosis) of fetal congenital adrenal hyperplasia at midgestation (Nimkarn and New, 2007). The placental deactivation of synthetic GCs is less efficient than that of natural GCs (Seckl, 1997). The receptors for both natural and synthetic GCs include intracellular GC receptors (GRs) and mineralocorticoid receptors (Seckl, 1997). According to a recently published preprint, the gene coding for GR, *NR3C1*, is expressed in human brain organoids from day 17 to day 158 of organoid development together with the whole molecular machinery that is needed for the response to GCs (Cruceanu et al., 2020). *NR3C1* expression is enriched in NPCs and GR can be identified in the nuclei of the cells forming neural rosettes (Cruceanu et al., 2020). Functionally, organoids are capable of upregulating GR-dependent gene expression upon acute exposure to dexamethasone (Cruceanu et al., 2020). Moreover, single-cell transcriptomic analysis indicates differential expression of genes responsible for fate determination in the developing brain, including HES6 and PAX6 upon dexamethasone treatment (Cruceanu et al., 2020). Gene ontology analysis showed enrichment for cell differentiation, head development, neurogenesis, neuron differentiation, and nervous system development in both neurons and NPCs, suggesting that GC exposure in the developing brain may interfere with neuronal differentiation (Cruceanu et al., 2020).

In addition to the direct effects of GCs on fetal brain development, they also affect the placenta. For instance, GCs decrease the transport of glucose to the fetal compartment of the placenta resulting in intrauterine growth restriction and upregulation of hypoxia-related genes at E12.5 in mice (Mueller and Bale, 2008). Thus, the effects of maternal stress may be attributed not only to the glucocorticoids but also to fetal response to nutrient deficiency and hypoxia (see below).

### Ethanol May Promote Premature Differentiation of Neural Precursor/Progenitor Cells in the Developing Human Brain

Maternal alcohol consumption can cause long-lasting deficits in fetal neurodevelopment leading to an increased risk for the development of psychiatric disorders (Ross et al., 2015). These deficits, also known as fetal alcohol spectrum disorder, have a global prevalence of 0.15% (Popova et al., 2017). Alarmingly, this number is 2.6 times higher in Europe suggesting a higher burden for the healthcare system (Popova et al., 2017). The severity of symptoms can vary from subtle neurobehavioral abnormalities to structural changes, including microcephaly (Ross et al., 2015). In the most extreme scenario, continuous drinking throughout pregnancy or binge drinking can cause fetal alcohol syndrome, which is characterized by both physical and neurobehavioral abnormalities (Ross et al., 2015). Importantly, ethanol, as a small molecule, readily crosses biological membranes, thus reaching the fetal brain. Recent data on primary human NPCs and NPCs-derived immature neurons suggests their particular vulnerability to increasing ethanol concentrations through the induction of apoptosis by alternative splicing of MCL-1, a member of the BCL2 protein family (Donadoni et al., 2019). Continuous exposure to ethanol (50 mM, 23 mg/dl) for 20 days starting from day 10 of differentiation, induces apoptosis in cerebral organoids and leads to a reduction of SOX2-positive cells while increasing the Tuj1-positive cell number (Zhu et al., 2017). Thus, NPCs either undergo cell death or prematurely differentiate into neurons upon ethanol exposure. Among the genes that were deregulated by ethanol exposure were cell adhesion molecules (Zhu et al., 2017). This observation has been functionally validated by attenuated neurite outgrowth in sliced organoids exposed to ethanol (Zhu et al., 2017).

Another study focused on acute ethanol exposure for 6 h of 2 month old cerebral organoids, where apoptosis was induced at ethanol concentrations of 115 mg/dl. This concentration is comparable to the blood ethanol concentration upon binge drinking (80 mg/dl, corresponding roughly to four glasses of wine over less than 2 h) (Arzua et al., 2020). In addition to apoptosis induction, the authors noticed ultrastructural changes, such as abnormal mitochondria cristae and glycogen foci (Arzua et al., 2020). The abnormal morphology of mitochondria was accompanied by functional changes in energy metabolism (Arzua et al., 2020). More changes were found at the transcriptional level with microarray analysis showing a deregulation of groups of genes involved in cell proliferation, nervous system development, and neurological diseases (Arzua et al., 2020). Given the complexity of cell type composition in cerebral organoids at 2 months of differentiation (Lancaster et al., 2013), these results provide an interesting starting point for further experiments on cell type-specific events.

### Nicotine, Cannabis, and Cocaine Interfere With Neurogenesis, as Seen in Brain Organoids

Prenatal nicotine exposure has been related to long-lasting changes in the developing brain (Faa et al., 2016). Specifically, it may affect neurogenesis, as seen in mice (Aoyama et al., 2016). Epidemiological reports indicate 15% of pregnant women smoke during pregnancy in the United States (Oh et al., 2017). Thirteen percent of newborns in the United Kingdom were exposed to environmental tobacco smoke and 36% to maternal smoking antenatally in 2000–2001 (Ward et al., 2007). Nicotine is well-known to cross the placenta and enter the fetal circulation in humans (Luck et al., 1985). In a recent study Wang and colleagues applied nicotine to brain organoids for 5 days during the early stages of neuroepithelial expansion and found that nicotine induced cell death as well as an increased proportion of Tuj1-positive cells suggesting premature differentiation of NPCs (Wang et al., 2018). Additionally, the expression of forebrain markers PAX6 and FOXG1 was depleted (Wang et al., 2018). Nicotine exposure at a later time point in organoid development resulted in the specific depletion of TBR1-positive layer VI neurons whereas the proportion of CTIP2-positive layer V neurons was increased (Wang et al., 2018). These findings indicate that nicotine exposure in critical periods of fetal brain development may disrupt proper cortical layering (Wang et al., 2018).

Like ethanol and nicotine, the major psychoactive component of cannabis, Δ-9-tetrahydrocannabinol (THC), readily crosses the placenta and enters the fetal circulation as seen in pregnant rats (Hutchings et al., 1989). However, it does not induce major developmental defects in the fetus (Warner et al., 2014). Its perceived safety leads to 1–5% of pregnant women using it in the general population with this number reaching 16.5% in pregnant adolescents (15–17 years old) (data reported for France and the United States) (Saurel-Cubizolles et al., 2014; Warner et al., 2014). However, mild longitudinal effects of this drug should not be overlooked. Epidemiological data indicate alterations in certain aspects of executive and visuoperceptual functioning in the adolescent offspring of mothers using cannabis during pregnancy (Huizink, 2014). Animal studies demonstrate protracted behavioral deficits in rat pups that were prenatally exposed to the synthetic cannabinoid receptor agonist WIN 55,212–2 (Mereu et al., 2003). Recent studies in cerebral organoids found that THC induced relative upregulation of PAX6 while downregulating neuronal markers CTIP2 and Tuj1 (Ao et al., 2020). Additionally, the expression of cannabinoid receptor CB1 was downregulated in organoids exposed to THC (Ao et al., 2020). CB1 is normally expressed in the embryonic and fetal brain (Díaz-Alonso et al., 2012; Nowakowski et al., 2017) and is known to be involved in the regulation of the balance between proliferation and neurogenesis in different brain areas, including cerebral cortex (Díaz-Alonso et al., 2012) consistent with the findings in cerebral organoids.

Cocaine is the third most commonly used substance and is being taken by 0.3% of women above the age of 12 in the United States (Ross et al., 2015). The European Monitoring Center for Drugs and Drug Addiction reports that 0.2–2.3% of adults (15–64 years old) used cocaine in 2018 in different countries, with the highest burden for Spain and the United Kingdom (EMCDDA, 2018).^1^ Cocaine is considered as a teratogen for the developing fetus, thus presenting a major concern for the healthcare system (Ross et al., 2015). It can be detected in the human placenta at PCW10 (Joya et al., 2010) and in the amniotic fluid at PCW14 (Apple and Roe, 1990). Cocaine is highly lipophilic and crosses the BBB, and accumulates in the adult brain (Farrar and Kearns, 1989) suggesting a similar effect in the fetus. Rodent and non-human primate studies have shown that maternal cocaine intake during the vulnerable window of neurogenesis causes structural abnormalities in the neocortex of the offspring (Lidow et al., 2001; Lidow and Song, 2001; Lee et al., 2011). Cytochrome P450 (CYP450) enzymes, which are the first to metabolize cocaine in the neural cells, were shown to have a causal role in the disturbance of neurogenesis in rats, where they induce reactive oxygen species (ROS)-dependent oxidative endoplasmic reticulum (ER) stress (Lee et al., 2008). The expression of CYP450 enzymes varies between species, making brain organoids an attractive model system for recapitulating human-specific abnormalities of corticogenesis upon cocaine exposure (Lee et al., 2017). Indeed, a dorsal forebrain organoid model identified expression of two members of CYP3A P450 subfamily, CYP3A5 and CYP3A43 (Lee et al., 2017). In the same study, periodic application of cocaine during the proliferative phase of organoid development resulted in the accumulation of ROS that was accompanied by the inhibited proliferation of NPCs and their premature differentiation (Lee et al., 2017). Finally, the neuronal output was reduced in cocaine-treated organoids (Lee et al., 2017). Knockdown of CYP3A5 reversed the pathological phenotype induced by cocaine (Lee et al., 2017). Interestingly, CYP3A enzymes are shown to metabolize ∼50% of all currently used therapeutic drugs suggesting their potential involvement in neocortical abnormalities caused by other drugs (Lee et al., 2017). In addition to the direct effects of cocaine on the fetal brain, it causes severe vasoconstriction in the mother leading to reduced blood flow in the placenta (Farrar and Kearns, 1989). This, in turn, results in restricted fetal growth and may also add fetal hypoxia to the compound effect of cocaine use by a pregnant woman (Farrar and Kearns, 1989).

## The Effects of Hypoxia on Neurogenesis are Time- and Dose-Specific

Oxygen pressure in the umbilical vein blood of the developing fetus at midgestation is half of the arterial blood of the mother (22–32 vs. 80–100 mmHg) showing that a hypoxic environment is natural for the developing fetus (Soothill et al., 1986). While physiological hypoxia is necessary for normal fetal development (Giaccia, 2004), several maternal factors may alter the delicate oxygen balance. Among them are certain medications, drugs, and high altitude (Jensen and Moore, 1997). Irrespective of the cause, the effects of hypoxia on fetal brain development depend on the severity of exposure and time window during development and vary between brain regions. The differential vulnerability of the fetal brain to hypoxia at different stages of development is recapitulated in organoids (Boisvert et al., 2019; Daviaud et al., 2019; Paşca et al., 2019). Indeed, hypoxic exposure in 10-day old brain organoids significantly reduces cortical marker expression (i.e. FOXG1, CTIP2, TBR1) later in the organoid development (Boisvert et al., 2019). In contrast, cortical spheroids exposed to hypoxic insult at day 75 of differentiation show mild changes, which are restricted to a specific cell type, the IPCs (Paşca et al., 2019). While taking into account different organoid protocols employed in these studies, we may roughly correlate these two time points with the development of the human brain at the early neuroepithelial stage and at midgestation (Paşca et al., 2019; Benito-Kwiecinski et al., 2021), respectively.

In addition to altered cortical marker gene expression, hypoxia induced apoptotic program in 10 day old brain organoids (Boisvert et al., 2019) (Figure 2C’). Considering that at this step of differentiation cerebral organoids consist almost exclusively of NECs (Benito-Kwiecinski et al., 2021), induction of apoptosis may be attributed to this cell type. Therefore, reduced cortical marker expression may be a secondary effect to the loss of NECs. Both apoptotic program induction and loss of cortical marker expression could be partially rescued by treatment with the antibiotic minocycline (Boisvert et al., 2019). Interestingly, minocycline has shown potential for the treatment of some neurodevelopmental diseases (Zhu et al., 2014; Mattei et al., 2017). Indeed, its administration to murine pups presented with maternal immune activation rescues the behavioral phenotype by normalizing the inflammatory status of microglial cells (Zhu et al., 2014; Mattei et al., 2017). However, this antibiotic is prohibited in pregnancy starting from PCW18 due to fetal toxicity related to skeletal development (Bevelander et al., 1960) and may thus have a limited potential to be used in humans.

Another study investigated the response to hypoxia in 4 week old cerebral organoids, where an immediate effect on apoptosis and DNA damage could be seen in the VZ-like area (Daviaud et al., 2019) (Figure 2C’’). The authors report a decreased number of TBR2-positive IPCs and FAM107A-positive oRGs 14 days after hypoxic exposure. Furthermore, the authors show alterations in the VZ-like zone, including the persistent loss of the proliferation marker Ki-67 and a shift in cleavage plane angles toward oblique or vertical suggesting self-renewal of vRG cells at the expense of indirect neurogenesis (Daviaud et al., 2019).

Interestingly, according to a study by Pasca and colleagues cell type-specific effects of hypoxia differed in 75 day old cortical spheroids from those at 4 weeks as reported by Daviaud and colleagues. Pasca and colleagues found that the number of RG cells marked by PAX6 was not altered upon hypoxic exposure (1% O2, 48 h) (Paşca et al., 2019). In contrast, they noticed specific depletion of TBR2-positive IPCs (Paşca et al., 2019) (Figure 2C’’’). RNA sequencing revealed the correlation between genes related to hypoxia and unfolded protein response (UPR), which marks ER stress (Paşca et al., 2019). The specific vulnerability of IPCs to ER stress was confirmed by loss-of-function and rescue experiments pointing toward a causal relationship between ER stress and IPCs depletion (Paşca et al., 2019). The depletion of IPCs was found to be caused by an increase in cell cycle exit (p27-positive cells), rather than induction of apoptosis (Paşca et al., 2019). ER stress and cell cycle exit in IPCs was followed by an increase in CTIP2-positive neurons suggesting premature differentiation of IPCs driven by UPR (Paşca et al., 2019).

In summary, brain organoids show differential vulnerability of particular cell types to hypoxic exposure applied at different time points of differentiation (Figure 2C). When applied early in the course of differentiation, hypoxia causes apoptosis in the VZ followed by potentially secondary effects on the numbers of basal progenitors (IPCs and oRGs) (Daviaud et al., 2019) and reduced expression of cortical markers (Boisvert et al., 2019). In contrast, hypoxic exposure at a later stage of organoid development leads to the loss of proliferative capacity in IPCs due to upregulation of UPR without affecting vRGs (Paşca et al., 2019).

## Brain Organoid Models Have Started to Reveal Cellular and Molecular Mechanisms of Environmental Insults

In the past years, brain organoids have been increasingly used for modeling the effects of environmental adversities on human neurogenesis (Table 2). Although organoid protocols differ in their speed of development, the majority of them are enriched for NPCs, particularly for vRGs, until day 50 of differentiation. Accordingly, the majority of the environmental insults listed in the Table 2 target these cells. Interestingly, environmental insults on vRGs often lead to either their death or premature differentiation resulting in the secondary loss of other NSCs and neurons. It was recently shown that murine vRGs have differential sensitivity to the environmental factors as a function of developmental age (Telley et al., 2019). Murine vRGs progress from an “introverted” state, when the cells do not express receptors to sense their surrounding at E12, to an “extraverted” state, when the cells are capable of responding to environmental cues later in development (Telley et al., 2019). In light of this finding, it would be interesting to analyze progressive changes in the transcriptome of vRGs within human brain organoids in order to decipher their sensitivity to the environmental factors.

**TABLE 2:** Summary of the neurogenic defects induced by environmental adversities modeled in brain organoids.

Group of environmental adversity	Environmental adversity	Publication	Organoid protocol	Regional identity, age of the organoid at the start of experiment	Major findings
Viral infections with vertical transmission	Zika virus	Gabriel et al. (2017)	Gabriel et al. (2017)	Brain, Day 9	Infection of NPCs followed by either apoptosis or premature differentiation due to defect in centriole assembly
		Dang et al. (2016)	Lancaster et al. (2013)	Cerebral, Day 10	Restricted growth
					TLR3 mediates transcriptional dysregulation of apoptosis and regulators of neurogenesis
		Qian et al. (2016)	Qian et al. (2016)	Cortical, Day 14 or 80	Infection in NPCs, including oRGs, as well as in IPCs and immature neurons
					Disrupted proliferation in the VZ-like areas
					Decreased neuronal output and increased size of ventricle-like cavities
		Watanabe et al. (2017)	Watanabe et al. (2017)	Cerebral, Day 21	Restricted growth
					Activation of innate immune response promoting cell death
		Albanese et al. (2020)	Lancaster et al. (2013)	Cerebral, Day 21	Restricted growth
					Decreased number of VZ-like areas
					Reduced number of vRGs accompanied with reduced neuronal output
		Wells et al. (2016)	Wells et al. (2016)	Cerebral, Day 24	Knockout of AXL does not protect VZ-like areas from viral infection and apoptosis
		Cugola et al. (2016)	Lancaster et al. (2013)	Cerebral, Day 28	Disruption of proliferative zones
					Decreased neuronal number and increased apoptosis
		Garcez et al. (2016)	Lancaster et al. (2013)	Cerebral, Day 35	Restricted growth
		Yoon et al. (2017)	Qian et al. (2016)	Cortical, Day 45	Disrupted apical polarity complex in vRGs, disrupted adherens junctions leading to premature differentiation
	Cytomegalovirus	Brown et al. (2019)	Lancaster et al. (2013) with modifications	Cerebral, Day 0	Decreased cellularity
					Regions of necrosis and cysts
					Disrupted VZ-like areas and radial scaffold
		Sun et al. (2020)	Lancaster et al. (2013) with modifications	Cerebral, Day 30	Restricted growth
					Decreased proliferation in the VZ-like areas
					Increased apoptosis adjacent to the VZ-like areas
					PDGFRa and EGFR are potential viral entry receptors
					Infection in TBR2-positive IPCs
					Decreased neuronal output
					Upregulated immune response and downregulated metabolism-related gene expression
		Cosset et al. (2015)	Preynat-Seauve et al. (2009)	Engineered neural tissue, age not reported	Upregulated lipid metabolism and inflammation-related genes
					Infection in doublecortin-positive newborn neurons but not in PAX6-positive NPCs
		Qiao et al. (2020)	Lancaster et al. (2013) with modifications	Cerebral, Day 15	Decreased expression of SOX2 and Nestin
					Decreased thickness of CP-like structures and decreased expression of neuronal markers
		D’Aiuto et al. (2019)	D’Aiuto et al. (2019)	Brain, age not reported	Infection in MAP2-positive neurons with formation of neuronal syncytia
		Rybak-Wolf et al. (2021)	Lancaster et al. (2017)	Cerebral, enriched for dorsal forebrain cellular identities, Day 60	Infection in different cell types with highest viral load in NPCs
					Cell type-specific changes in transcriptional profile
					Global elongation of poly(A) tails and preferential use of distal 3’UTR in mRNA molecules
	SARS-CoV-2	Ramani et al. (2020)	Gabriel et al. (2016), a modification of Lancaster et al. (2013)	Cerebral, Day 15 or 60	Little to no infection in organoids inoculated with the virus on Day 15 of differentiation, significant infection in Day 60 organoids
					Infection in Tuj1-positive neurons
					Apoptotic neuronal cell death due to aberrant tau localization
		Zhang et al. (2020)	Lancaster et al. (2013)	Cerebral, Day 35	Infection in Nestin-positive NPCs and Tuj1-positive neurons
					Productive infection of the organoid
		Mesci et al. (2020)	Trujillo et al. (2019)	Cortical, Day 52	Infection and increased apoptosis rate in Nestin-positive NPCs, MAP2-positive neurons and GFAP-positive astrocytes
					The phenotype is reversed by sofosbuvir
		Song et al. (2020)	Lancaster et al. (2013)	Cerebral, Week 9	Productive infection in SOX2-positive NPCs and MAP2-positive neurons but not in GFAP-positive astroglia
					ACE2 protein localization to MAP2-positive neurons and close to the VZ-like cavities
					Overall increased apoptosis rate within the organoid irrespective of the infection status of the cell
					Hypermetabolic state of the infected cells and overall downregulation of catabolic processes
		Andrews et al. (2021)	Pollen et al. (2019)	Cortical, Week 5, 10, 16, or 22	Infection in double-GFAP,AQP4-positive astrocytes and rare infection of NeuN-positive neurons at Week 22
					No infection in SOX2-positive NPCs at Week 5 and 10
					No ACE2 protein expression detected
		Jacob et al. (2020)	Qian et al. (2016)	Cortical, age not reported	Infection in doublecortin-positive neurons
Maternal stress, medication, and substance use	Glucocorticoids (dexamethasone)	Cruceanu et al. (2020)	Lancaster et al. (2013)	Cerebral, Day 45	Cerebral organoids express the molecular machinery for response to glucocorticoids starting from Day 17 of differentiation
					GR expression is enriched in NPCs
					Altered expression profile indicates that dexamethasone interferes with neuronal differentiation
	Ethanol	Zhu et al. (2017)	Zhu et al. (2017), an organ-on-chip modification of Lancaster et al. (2013)	Cerebral, Day 10	Apoptosis induction
					Decreased SOX2-posivive NPC number
					Increased Tuj1-posivive neuron number
					Decreased expression of cell adhesion molecules
		Arzua et al. (2020)	Lancaster et al. (2013)	Cerebral, Day 60	Apoptosis induction in NeuN-positive neurons but not in S100B-positive astrocytes
					Altered energy metabolism and mitochondrial function
					Altered gene expression profile including genes related to neurodevelopment and neurological diseases
	Nicotine	Wang et al. (2018)	Wang et al. (2018), an organ-on-chip modification of Lancaster et al. (2013)	Cerebral, Day 11	Induction of apoptosis
					Increased proportion of Tuj1-positive neurons indicating premature differentiation
					Decreased expression of forebrain markers PAX6 and FOXG1
	Cannabis	Ao et al. (2020)	Ao et al. (2020), a microfluidic modification of Lancaster et al. (2013)	Cerebral, Day 3	Increased PAX6-positive NPCs number and increased thickness of VZ-like areas indicating increased proliferation of NPCs
					Decreased expression of neuronal markers Tuj1 and CTIP2
					Downregulated CB1 expression
	Cocaine	Lee et al. (2017)	Lee et al. (2017)	Cortical, Day 32	Decreased PAX6-positive NPCs number
					Increased migration of BrdU-positive neurons indicating premature differentiation
					Increased ROS formation
Fetal hypoxia	Fetal hypoxia	Boisvert et al. (2019)	Boisvert et al. (2019)	Brain, Day 10	Induction of apoptotic program
					Decreased expression of cortical markers FOXG1, CTIP2, TBR1
					Both the induction of apoptotic program and decreased cortical marker expression may be reverted by minocycline
		Daviaud et al. (2019)	Lancaster et al. (2013)	Cerebral, Day 28	Immediate cell death in VZ-like areas and increased CTIP2-positive neuron number
					Delayed decrease in TBR2-positive IPCs and FAM107A-positive oRGs
					Self-renewal divisions in aRGs at the expense of indirect neurogenesis
		Paşca et al. (2019)	Sloan et al. (2018)	Cortical spheroid, Day 75	No cell death observed
					PAX6-positive NPCs number not altered
					Decreased number of TBR2-positive IPCs due to premature cell cycle exit and differentiation

Finally, the diversity of the existing protocols (Table 2) requires particular attention when comparing and extrapolating results from other studies and designing experiments. The following prerequisites may provide a roadmap for the experimental design when modeling environmental adversities on human neurogenesis with brain organoids:

1. Brain organoids should recapitulate the main stages of (early) brain development in a timely manner and display characteristic cytoarchitectural features of the developing brain. The benchmarking of the organoid differentiation should be clearly reported in the publication;
2. Environmental insults should have a form that reaches the fetus *in utero* meaning that the fetus should not be protected from it through one of the previously discussed barriers (i.e. placenta and blood-brain interfaces);
3. In order to be able to draw solid conclusions, the experimental design should include several cell lines, several batches of organoid differentiation and a sufficient number of technical replicates within each batch. For neurodevelopmental phenotypes with differential gender penetrance, both male and female PSC lines should be included in the study.

## Technological Advances Drive Progress in Brain Organoid Research and Increase Interpretability Across Studies

### Recent Developments Aim at Overcoming Limitations of Organoid Models

Brain organoids are a promising model system, but do not fully recapitulate human brain development *in vivo*. First, they lack the cells not originating from neuroectoderm, such as those of the vasculature and microglia (Khakipoor et al., 2020). Specifically, the vasculature is important for the proper modeling of fetal brain hypoxia with brain organoids. Brain vasculature is known to have a pivotal role in switching the NPCs from proliferative state to neurogenic one by resolving hypoxia (Lange et al., 2016). The first attempts to recapitulate interactions between brain organoid tissue and vascular cells *in vitro* were made in 2018 (Pham et al., 2018) and further advanced since then (Cakir et al., 2019; Shi et al., 2020). Vasculature-like tubes within brain organoids express tight junction proteins and exhibit BBB-like functions (Cakir et al., 2019). The use of such organoids will advance the neurotoxicity research. Additionally, the introduction of vascular-like cells helped to reduce the size of the hypoxic core in the organoids (Cakir et al., 2019) which is a valuable improvement in light of the recent finding that glycolytic and ER stress impairs cell subtype specification in brain organoids (Bhaduri et al., 2020).

Microglial cells are brain resident macrophages that originate from the yolk sac (Utz et al., 2020). They are likely important players in translating environmental adversities to abnormalities in fetal brain development. To date, the majority of studies have focused on the role of environmental factors in shaping synapse pruning and circuit maturation by microglia (Salter and Stevens, 2017). However, microglial cells can be found in the human forebrain as early as PCW4.5 where they reside within and next to the VZ (Monier et al., 2007). Early microglia plays an active role in regulating neurogenesis in the primate neocortex (Cunningham et al., 2013). Moreover, human microglia at PCW11 starts acquiring a more mature phenotype including the activation of environment-sensing programs (Kracht et al., 2020). It is, therefore, likely that microglial cells can sense the local environment starting from the midgestation and adapt their interactions with the NSCs accordingly. Human brain organoids can be colonized with induced PSC-derived microglial progenitors, which differentiate within the organoid (Abud et al., 2017; Fagerlund et al., 2020). We suggest that in order to model environmental adversities with an inflammatory component more faithfully *in vitro*, the use of such brain organoid-microglia co-cultures is beneficial.

As mentioned before, insufficient oxygen and nutrient supply due to large distances not overcome by diffusion may impact differentiation trajectories of the NSCs within the organoid (Bhaduri et al., 2020). To overcome this limitation, several groups have proposed to maintain the organoids in slice cultures (Qian et al., 2020; Giandomenico et al., 2021). Particularly, slicing of the organoids was shown to sustain the neurogenic capacity of the NSCs for longer, thus allowing the generation of distinct cortical layers (Qian et al., 2020). Alternatively, the organoids may be cultivated on the bio-compatible microfilaments allowing for higher area-to-volume ratio (Lancaster et al., 2017). Further improvement may be achieved by using organ-on-chip approaches that allow better control over the biochemical environment of the organoid. Organ-on-chip methods have been applied to study the effects of nicotine (Wang et al., 2018), cadmium (Yin et al., 2018), and ethanol (Zhu et al., 2017) exposure on brain organoids.

We suggest that modeling environmental effects on fetal brain development would benefit from combining placental barrier, blood-brain interfaces and brain organoids in an *in vitro* setting. This would enhance our understanding of the mechanisms of viral infections and could serve as a versatile platform for developmental neurotoxicity testing. This might be a possibility in the near future since *in vitro* models of the placental barrier (Blundell et al., 2016; Muoth et al., 2016), the BBB (Wang et al., 2017) and the blood-CSF barrier (Monnot and Zheng, 2012; Pellegrini et al., 2020b) have been developed recently.

### The Scalability of Brain Organoids Poses a Variety of Challenges and New Opportunities

One of the major assets of brain organoids as a model system is their scalability. Indeed, depending on the protocol, one researcher can maintain hundreds of organoids in parallel and automation approaches that are currently being developed could further allow high-throughput experiments (Sirenko et al., 2019; Renner et al., 2020). However, the scaled generation of organoids requires appropriate readout and data analysis options. These include 3D imaging as well as a variety of omics approaches and large-scale genetic screens.

Tissue clearing protocols have recently been adapted for brain organoids allowing immunohistochemistry to be combined with whole-mount imaging (Albanese et al., 2020; Renner et al., 2020). Whole-mount microscopy allows the unbiased estimation of cell counts within the organoid and evaluation of tissue cytoarchitecture (Albanese et al., 2020). However, the organoids lack a stereotyped anatomical arrangement which precludes “tissue atlas”-based volumetric analysis (Albanese et al., 2020). Instead, “atlas-free” analysis strategies must be employed, which rely on artificial neural networks (Albanese et al., 2020). In addition to the analysis of marker expression, the scaled generation of organoids requires careful automatic monitoring from bright field images or the use of reporter cell lines to closely follow organoid differentiation trajectories without endpoint analysis, for example accounting for unsuccessful differentiation (Bagley et al., 2017; Kegeles et al., 2020).

The development of organoid generation protocols coincided with the evolution of single-cell transcriptomics. This overlap resulted in a series of works comparing single-cell signatures of neural cells within brain organoids to those in the developing human brain (Camp et al., 2015; Pollen et al., 2019; Velasco et al., 2019; Bhaduri et al., 2020; Tanaka et al., 2020). Omics approaches, and especially single-cell omics approaches, require tailored data analysis toolboxes (Lähnemann et al., 2020). Additionally, in order to characterize cellular identity and function in a holistic manner, the combination of several modalities at the single-cell level, while challenging, is becoming increasingly feasible. We have recently developed a method to combine calcium imaging with single-cell transcriptomics in the developing human neocortex revealing changes in physiological features alongside transcriptomic changes as neurons differentiate (Mayer et al., 2019). Another modality, that is often combined with single-cell transcriptomics, is single-cell epigenomics (Amiri et al., 2018). Recently, cell type-specific chromatin accessibility analysis in long-term organoid culture revealed an epigenetic switch resembling the transition from pre- to postnatal development in humans (Trevino et al., 2020). Finally, a proof-of-principle study showed that bulk proteomic approach can be applied to resolve the effects of a psychedelic analogue of serotonin on brain organoids (Dakic et al., 2017). The integration of several data modalities also requires specific data analysis techniques. These unique challenges can be approached from different perspectives, including facilitation of data analysis with artificial intelligence-based approaches (Badai et al., 2020).

Altogether, the advances in single-cell omics could allow to decipher cell type-specific events upon the exposure to an environmental factor. Indeed, single-cell transcriptomics has recently helped to reveal sex-specific changes in the neuronal development in mice upon maternal immune activation (Kalish et al., 2021). Importantly, environmental exposures are likely to have long-lasting effects on developmental outcomes also through epigenetic mechanisms (Schaevitz and Berger-Sweeney, 2012; Svrakic et al., 2013). Therefore, we propose that analyzing how environmental exposures affect epigenetic features of specific cell types together with single-cell transcriptomics will be leading to new insights.

The epigenetic component is likely one of the players defining the effect of environmental exposures on the fetal neurodevelopment. Another side of the coin is the genetic predisposition for a neurodevelopmental disorder. Together, genetic predisposition followed by an environmental adversity may increase the risk to develop a neurodevelopmental disorder. This idea, the so-called “double-hit” hypothesis, may explain the development of complex disorders like autism spectrum disorder (ASD) (Schaevitz and Berger-Sweeney, 2012; Schaafsma et al., 2017). To study the genetic component of ASD and other neurodevelopmental disorders and its interaction with the environmental adversity, genetic screening applications can be used. The first step in this direction was made by employing CRISPR–lineage tracing at cellular resolution in heterogeneous tissue (CRISPR-LICHT) technology to study genetic risk factors for primary microcephaly (Esk et al., 2020). Here two complementary techniques, namely inducible CRISPR-Cas9-mediated gene editing and dual DNA barcoding were combined to allow lineage tracing from individual embryonic stem cells used for organoid generation (Esk et al., 2020). This study thus provides the foundation for single-cell tracing of the proliferative and neurogenic capacity of various NSCs with different genetic backgrounds in the future (Esk et al., 2020).

## Discussion

Recently, organoid research has allowed increasing insights into the effect of environmental insults on brain development on a cellular and molecular level. In light of the new developments in organoid research both at the level of organoid protocols (such as inclusion of vasculature, microglia, and bioengineering) and readouts (multimodal omics approaches, physiological readouts, whole-mount stainings), we expect even greater impacts in the years ahead.

For the future, we suggest that it will be important to integrate the perspectives on environmental programming of different disciplines further to generate breakthroughs in this research field that has widespread medical and societal implications. We propose that this may be achieved by tighter collaborations between scientists working on *in vitro* models, on animal models, and performing studies in humans from a systems neuroscience and medical point of view.

## Footnotes

1
https://www.emcdda.europa.eu/data/stats2018/gps_en.

## Glossary

NSC: neural stem cell
PCW: postconceptional week
PSC: pluripotent stem cell
NEC: neuroepithelial cell
NPC: neural precursor/progenitor cell
vRG: ventricular radial glia
IPC: transit-amplifying/intermediate progenitor cell
oRG: outer radial glia
iSVZ: inner subventricular zone
oSVZ: outer subventricular zone
tRG: truncated radial glia
IZ: intermediate zone
SP: subplate
CP: cortical plate
MZ: marginal zone
TGF-β: tumor growth factor β
BMP: bone morphogenetic protein
BBB: blood-brain barrier
CSF: cerebrospinal fluid
ChP: choroid plexus
ToRCH pathogens: toxoplasmosis, rubella, cytomegalovirus, herpes simplex virus, and others
AQP1: aquaporin 1
TLR3: Toll-like receptor 3
AXL: AXL receptor tyrosine kinase
TYRO3: tyrosine-protein kinase receptor
MER: proto-oncogene tyrosine-protein kinase
TIM1: T-cell immunoglobulin and mucin domain 1 or hepatitis A virus cellular receptor 1
SOX2: (sex determining region Y)-box 2 protein
TBR1: T-box brain transcription factor 1
CMV: cytomegalovirus
PDGFRa: platelet derived growth factor receptor alpha
EGFR: epidermal growth factor receptor
TBR2: T-box brain transcription factor 2, alternative name EOMES
IE1: immediate early gene of cytomegalovirus
PAX6: paired box protein 6
HSV: herpes simplex virus
ACE2: angiotensin-converting enzyme 2
TMPRSS2: transmembrane protease, serine 2
GC: glucocorticoid
11βHSD2: 11β-hydroxysteroid dehydrogenase type 2
GR: glucocorticoid receptor
NR3C1: nuclear receptor subfamily 3 Group C member 1, gene coding for GR
HES6: Hes Family BHLH transcription factor 6
MCL-1: induced myeloid leukemia cell differentiation protein
BCL2: B-cell lymphoma 2 protein
FOXG1: Forkhead box G1 protein
CTIP2: alternative name BCL11B, B-cell lymphoma/leukemia 11B protein
THC: Δ-9-tetrahydrocannabinol
Tuj1: neuron-specific class III beta-tubulin
CB1: cannabinoid receptor type 1
CYP450: cytochrome P450
CYP3A: cytochrome P450 family 3 subfamily A
ROS: reactive oxygen species
ER: endoplasmic reticulum
UPR: unfolded protein response
ASD: autism spectrum disorder
CRISPR: clustered regularly interspaced short palindromic repeats
CRISPR-LICHT: CRISPR-lineage tracing at cellular resolution in heterogeneous tissue
